# Morphological and Mechanical Properties of Electrospun Polycaprolactone Scaffolds: Effect of Applied Voltage

**DOI:** 10.3390/polym13040662

**Published:** 2021-02-23

**Authors:** L.A. Can-Herrera, A.I. Oliva, M.A.A. Dzul-Cervantes, O.F. Pacheco-Salazar, J.M. Cervantes-Uc

**Affiliations:** 1 Departamento de Física Aplicada, CINVESTAV-IPN, Unidad Mérida, Carretera Antigua a Progreso Km. 6, Cordemex, C.P. 97310 Mérida, Yucatán, Mexico; oliva@cinvestav.mx; 2 Instituto Tecnológico Superior de Calkiní en el Estado de Campeche, Av. Ah Canul S/N por Carretera Federal, C.P. 24900 Calkiní, Campeche, Mexico; maadzul@itescam.edu.mx (M.A.A.D.-C.); ospacheco@itescam.edu.mx (O.F.P.-S.); 3 Unidad de Materiales, Centro de Investigación Científica de Yucatán, A.C. Calle 43 No. 130 × 32 y 34, Chuburná de Hidalgo, C.P. 97205 Mérida, Yucatán, Mexico; manceruc@cicy.mx

**Keywords:** polycaprolactone, electrospinning, voltage, morphological properties, mechanical properties

## Abstract

The aim of this work is to investigate the effect of the applied voltage on the morphological and mechanical properties of electrospun polycaprolactone (PCL) scaffolds for potential use in tissue engineering. The morphology of the scaffolds was characterized by scanning electron microscopy (SEM), atomic force microscopy (AFM), and the BET techniques for measuring the surface area and pore volume. Stress-strain curves from tensile tests were obtained for estimating the mechanical properties. Additional studies for detecting changes in the chemical structure of the electrospun PCL scaffolds by Fourier transform infrared were performed, while contact angle and X-ray diffraction analysis were realized for determining the wettability and crystallinity, respectively. The SEM, AFM and BET results demonstrate that the electrospun PCL fibers exhibit morphological changes with the applied voltage. By increasing the applied voltage (10 to 25 kV) a significate influence was observed on the fiber diameter, surface roughness, and pore volume. In addition, tensile strength, elongation, and elastic modulus increase with the applied voltage, the crystalline structure of the fibers remains constant, and the surface area and wetting of the scaffolds diminish. The morphological and mechanical properties show a clear correlation with the applied voltage and can be of great relevance for tissue engineering.

## 1. Introduction

Electrospinning allows exceptional versatility to produce fiber scaffolds with targeted properties [1]. Varying some electrospinning parameters, features of the morphology can be enhanced, resulting in better mechanical properties [2].

The fiber morphology is greatly influenced by numerous processing parameters in the electrospinning process, as has been widely reported [2,3,4,5]. The solution properties (surface tension, conductivity, polymer concentration, viscosity, etc.), process parameters (applied voltage, distance to the collector, flow rate), and ambient conditions (humidity and room temperature) are factors that affect the diameter and morphology of the fibers [4]. Although some solution parameters show dependence among them, the applied voltage does not [4]. Among all parameters, applied voltage is one of the most important for controlling the morphology of the nanofibers, without which the jet formation could not occur [5]. Referring to the applied voltage, at low values, the Coulombic forces are not enough to overcome the superficial tension of the polymer solution, which results in droplets/beads formation instead of fibers.

By increasing the voltage, the superficial tension and viscoelastic forces equilibrate, forming a charged jet and producing small fiber diameters. At higher applied voltages, the Coulombic forces overcome the viscoelastic forces, breaking the jet, and higher diameters are produced [5,6].

Several reports [7,8,9,10,11], have studied the effect produced by voltage applied on electrospun polycaprolactone (PCL) scaffolds, even though most of them are dependent to other parameters of the electrospinning process. Furthermore, authors report that the set of these variables could improve the morphological and mechanical properties of the scaffolds. Some authors affirm that the applied voltage favors the increment of the fiber diameter [7], pore size/volume [8], nanofiber roughness [9], and the tensile strength [10,11]; however, other authors affirm the opposite [12,13]. In addition, most of these reports indicate that the morphology of the scaffolds is caused by more than one variable during the electrospinning process, although in this work we assume that the morphology of electrospun PCL fibers can be controlled by modifying only the voltage. For example, Doustgani et al. [7] show an increase in the mean fiber diameter (MFD) of electrospun fibers by increasing the applied voltage from 10 to 22 kV and for the incorporation of nanohydroxyapatite (nHA), i.e., they do not show conclusive results that independently distinguish the effect of each parameter on the fiber diameter. In other works [11] of these authors, they demonstrated that the mechanical properties of electrospun PCL improved by increasing the applied voltage and the nHA concentration. Katsongiannis et al. [8] reported that an increase in applied voltage favors the formation of pores and increases the fiber diameter of the PCL scaffolds. They concluded that the pore formation is related to the increment in the PCL solution flow rate (to from 0.5 to 5 mL/h). Hassan et al. [9] found that fiber roughness is greatly influenced by the concentration and viscosity of the solution, as the applied voltage increases, but they do not reveal the effect of each variable separately on the characteristics of the scaffolds.

To overcome the limitations represented by the concentration and viscosity of the polymer solution related to the applied voltage, Baldino et al. [14] and Li et al. [15] suggested a new methodology called electrospinning assisted by supercritical-carbon dioxide (SC-CO_2_). Although this methodology could be considered as a good alternative for solvent-free polymer processing, Li et al. [15] concluded that more fundamental efforts must be made to understand and take advantage of the phase separation generated under supercritical conditions to know the effect of the variables.

Thus, from these results, is clear that further studies are required to clearly establish the effect of only the applied voltage on the morphological and mechanical properties of the electrospun PCL.

In this study, the effect of the applied voltage (10–25 kV) in the electrospinning process was evaluated on the features of PCL scaffolds. Fiber diameter, pore volume, fiber roughness, wetting, tensile strength, and elastic modulus are parameters studied. In order to observe the effect of the applied voltage, morphological (SEM, AFM, BET), and mechanical (stress strain curves) analysis were used. In addition, water contact angle and XRD were measured to analyze hydrophilicity and crystallization index, respectively, while FTIR was used to observe changes in the chemical structure on the electrospun PCL scaffolds.

## 2. Materials and Methods

### 2.1. Materials

PCL with 70,000–90,000 g/mol of molecular weight was used to fabricate the electrospun scaffolds. Methanol (MeOH, 99.9% purity) and chloroform (CHCl_3_, 99.8% purity) were used as solvents. The PCL and all solvents were acquired from Sigma-Aldrich (Saint Louis, MO, USA) and used as received.

#### Preparation of the PCL Electrospun

The PCL was magnetically stirred at 12% w/v in a mixture of CHCl_3_–MeOH (3:1 ratio) at room temperature (25 °C) for 4 h. A total of 8 mL of this solution was placed in a plastic syringe with a needle of 0.4 mm-diameter. A Nabond equipment was used for the horizontal electrospinning process, maintaining the chamber at relative humidity of 40–48% with silica gel and room temperature. To evaluate the effect of the applied voltage in the electrospinning process, different values were established based on preliminary experiments. The selected voltage values generate electrospun scaffolds with homogeneous fiber without beads in their structure. The flow rate of the PCL solution and the distance of collector were 1.5 mL/h and 12 cm, respectively. For low flow rates and short distances, fibers cannot be formed; while for higher flow rates and longer distances, non-homogeneous fibers and pores appear. The formed electrospun scaffolds were dried at room temperature in a vacuum oven for 24 h and stored in a vacuum glass desiccator until characterization.

### 2.2. Morphological Characterization of the PCL Electrospun Scaffolds

#### 2.2.1. Scanning Electron Microscopy

The obtained PCL scaffolds were analyzed in a JSM 6360LV scanning electron microscope (SEM). Images of different sites of the scaffolds were obtained at 2000× at 10–20 kV. The SEM images were analyzed by the Image PRO software and the mean diameter of fibers, their standard deviation, and statistics distribution were reported.

#### 2.2.2. Atomic Force Microscopy

The root mean squared roughness (*R_rms_*) was estimated from the images obtained with an atomic force microscope (AFM, Ambios Universal, CA, USA) using a Si-N cantilever in non-contact mode and the WSxM v5.0 develop 8.1 free software from Nanotec [16]. AFM images were taken with 512 pixels’ × 512 pixels of resolution.

#### 2.2.3. Surface Area and Total Pore Volume by BET Method

The surface area and the total pore volume (or porosity) of the PCL scaffolds were estimated by the adsorption/desorption isotherms obtained by the BET technique. The analysis was conducted at 77 K (−196 °C) in a Quantachrome Instruments equipment, model NOVA 1200e. The samples were degasified with nitrogen at 13.33 × 10^−5^ Pa at room temperature during 24 h for reducing impurities [17]. The quantity of the analyzed sample was ≈50 mg.

### 2.3. Physicochemical Characterization of the PCL Scaffolds

#### 2.3.1. Fourier-Transform Infrared Spectroscopy in Attenuated Total Reflectance (FTIR-ATR) Analysis

The PCL scaffolds were characterized by FTIR-ATR analysis using a ZnSe crystal. Spectra were obtained in a Nicolet 8700 equipment (Thermo Fisher Scientific Inc., Waltham, MA, USA) using 100 scans and 4 cm^−1^ of resolution in a wave number range from 4000 cm^−1^ to 650 cm^−1^.

#### 2.3.2. X-ray Diffraction (XRD) Analysis

The XRD analysis was performed to determine the crystalline structure of the electrospun PCL fibers as a function of the applied voltage. The analysis was performed in a diffractometer Siemens D5000, with step time of 6 s, step size of 0.04°, 34 kV, and 25 mA. In order to estimate the crystallinity index (%CI) of the polymer, the methodology proposed by Nara et al. was used [18]. Briefly, an amorphous area is estimated plotting a baseline, which joins the valleys and peaks of the diffractograms; subsequently, the crystalline zone is estimated from the area under the curve of the peaks [18]. The %CI value is determined by knowing the crystalline area A_c_, and the amorphous area *A_a_*, according to Equation (1).

(1) $$\%CI=\frac{A_{C}}{A_{C}+A_{a}}\times 100$$

#### 2.3.3. Contact Angle Analysis

The water contact angle of the materials was measured with the sessile drop method, using an optical goniometer Tantec Inc., and a precision needle (Hamilton, Inc., Morehead, KY, USA). For that, 5 μL of distilled water was poured on the surface at five different sites of the evaluated PCL scaffolds.

### 2.4. Mechanical Characterization of the Electrospun PCL Scaffolds

**Figure 1:**
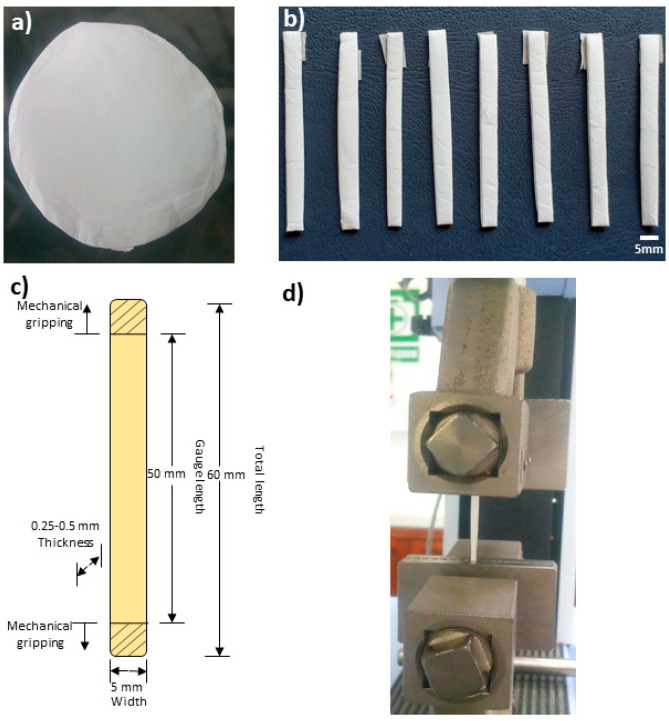
(**a**) Polycaprolactone (PCL) scaffold obtained by electrospinning, (**b**) tensile specimens according to the ASTM D822 standard, (**c**) specimen dimensions, and (**d**) holding the specimen in the tensile machine.

The scaffolds obtained by electrospinning at different voltages was mechanically tested in a universal testing machine Shimadzu AGS-X (Shimadzu, Kyoto, Japan) at room temperature using a 100 N-load cell and a crosshead speed of 10 mm/min. From the obtained electrospun fibers mat (see Figure 1a), eight rectangular samples were cut (see Figure 1b) and prepared according to the ASTM D882 standard [19]. Specimen size was 60 mm × 5 mm × 0.25–0.5 mm with a gauge length of 50 mm [20] (Figure 1c); the mechanical testing setup is shown in Figure 1d. The thickness of each specimen was measured with a Quick mini Mitutoyo micrometer. From the obtained stress-strain curves, the tensile strength, and the elastic modulus of each specimen was estimated.

The tensile strength of samples (*σ* = *F/A0*) was determined by the applied load (*F*) and the cross-sectional area (*A0*). The unitary deformation (*ε* = ∆*l/l0*) relates the change of displacement of the gripper (∆*l*) with the initial length of the sample (*l0*). As a consequence, the elastic modulus (*E* = σ/ε) can be calculated [21].

### 2.5. Statistical Analysis

The tests were performed in triplicate, for mechanical tests 8 samples were analyzed for each group. The mean value and standard deviation were obtained for all cases. In addition, a one-way analysis of variance (ANOVA) was done using the *Tukey* test, where a value of *p* ≤ 0.05 was considered significant.

## 3. Results and Discussion

### 3.1. Morphology of the Electrospun PCL Scaffolds

#### 3.1.1. Scanning Electron Microscopy

**Figure 2:**
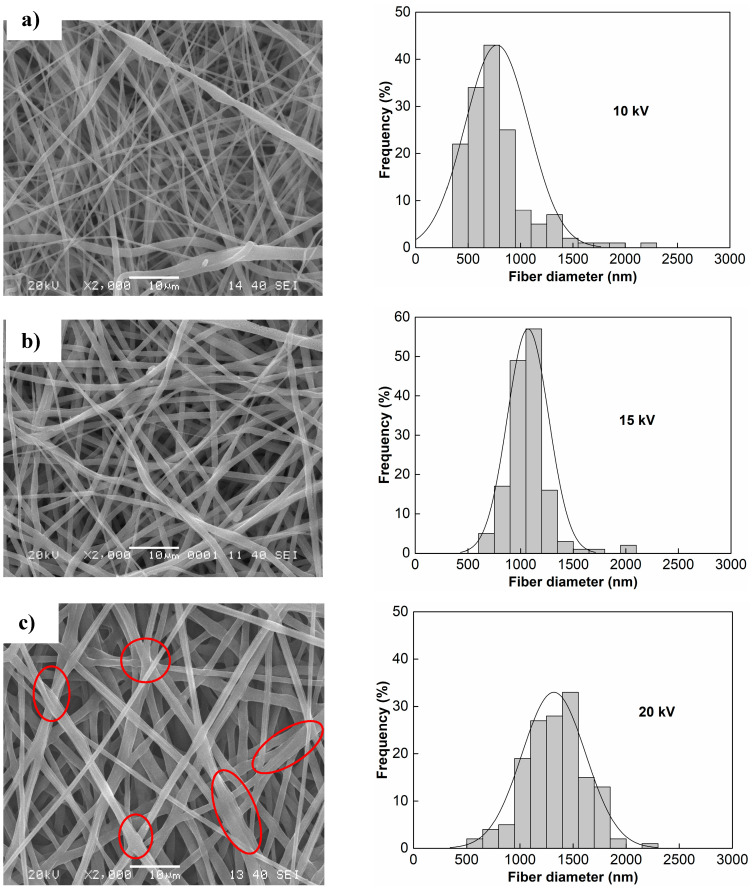

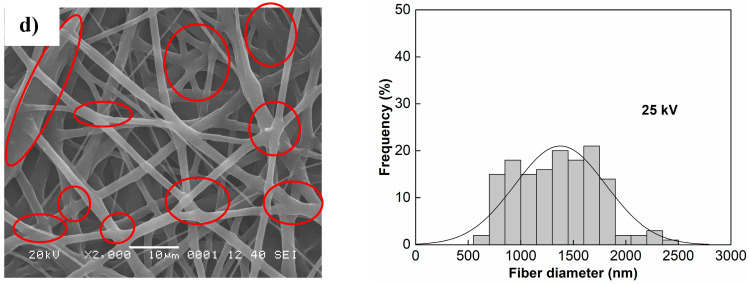
Micrographs (left-side) and fitted normal distribution curve (right-side) of the PCL fiber diameters obtained at applied voltages: (**a**) 10 kV, (**b**) 15 kV, (**c**) 20 kV, and (**d**) 25 kV.

On the left column of Figure 2 can be observed the micrographs of the electrospun PCL scaffolds obtained at different voltages, while on the right column of each micrograph, the corresponding diameter distributions and the fitted normal distribution curve are included.

As can be observed from the micrographs, the fibers show a homogeneous appearance, and no beads were detected for the different voltages. For tissue engineering applications, no beads in the PCL fibers are desirable; moreover, the fiber diameters need to be similar than the natural extra cellular morphology for promoting the growth of cells [3].

In this work, the relationship between voltage and fiber morphology are based on the SEM images and on the statistical analysis. In Figure 2, large pore size and branch-shaped fibers are observed when the voltage increases.

This can be explained as follows: high voltages induce multiple jets of electrospinning, reducing the electrostatic forces and stretching the fibers [22]; as a result, shrunk fibers are obtained. In addition, higher voltages (20 and 25 kV) produce points of contact between fibers (see closed regions in Figure 2c,d) and branched structures could influence the mechanical results [23], as we will discuss later.

**Figure 3:**
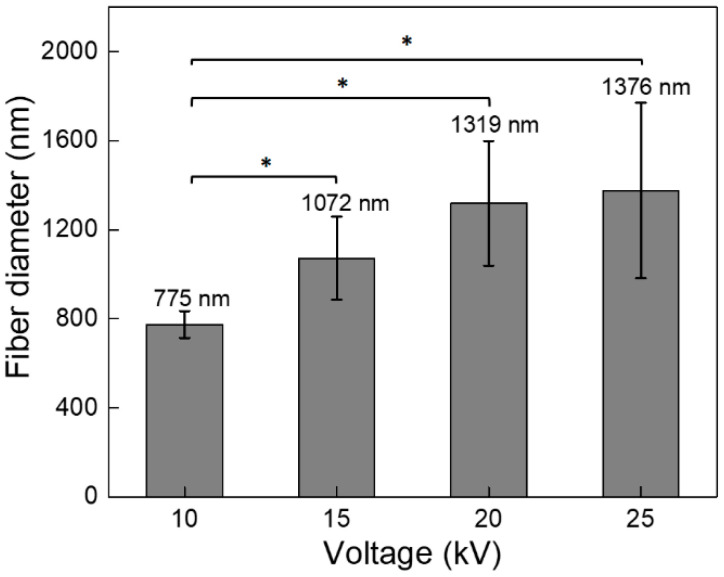
Average fiber diameter of scaffolds as a function of the applied voltage. The standard deviation is included. * Statistical changes were obtained by comparing the lowest values with 15, 20, and 25 kV.

An increase in the fiber diameter is generally observed when the voltage increases from 15 kV to 20 kV (Figure 3). Higher voltages produce acceleration towards the collector, leading to reduced flight time for stretching the jet prior to deposition, which allows obtaining fibers with larger diameter [24]. Baumgarten [25] studied the flow rate, voltage, and the jet of the polymer solution, reporting that the jet increases with the voltage for certain polymer solutions and optimal flow rate. This is desirable to establish the effect of each parameter on the final morphological features of the electrospun PCL [26].

From the statistical distribution of diameters can be seen similar shapes, except for the sample at 10 kV, which exhibited a skewed right distribution. However, as the voltage increases the average diameter increases and the distribution curve widens in a similar way to other reports for polymeric PCL scaffolds [27]. By the fiber morphology in the branch form, samples prepared with 25 kV are free of beads. This can be due to the solubility of PCL in chloroform [28] and the equilibrated volatility [29]. It is known that for each polymer-solvent system there exists a critical/equilibrium value and once the critical value is reached, bead-free materials are obtained. This equilibrium value depends largely on the intrinsic properties of the solution [29]. Kołbuk et al. [30] observed a decrement in the fiber diameter for a mixture of chloroform: dimethylformamide, but for a chloroform: methanol mixture (as in this work) an increase in the fiber diameter was obtained for higher voltages.

Figure 3 shows the average diameter values of fibers as a function of the applied voltage. An increasing voltage produces increments on the fiber diameter, from 700 to 1400 nm (see histograms in Figure 2). Furthermore, the analysis of variance indicates that the tendency is statistically significant by comparing the lowest value (10 kV) versus all other samples (15 kV, 20 kV, and 25 kV).

The results reported in this work are similar to those reported by Cipitria et al. [31] who increased the voltage from 13 kV to 20 kV for obtaining electrospun PCL scaffolds, and an increased fiber diameter from 600–700 nm to 1000–2000 nm.

The range of fiber diameters obtained in our work (700–1400 nm) is desirable for cell migration [32]. Badami et al. reported that fibers with small diameters (140 nm or less) could inhibit cell infiltration and proliferation, [32] given that reduced fiber diameter produces minor pore size of the scaffolds [33,34]. However, on the other hand, electrospun scaffolds whose diameter fiber is greater than 1600 nm exhibited a decrease in cell attachment and proliferation of fibroblast of electrospun PCL scaffolds [34].

#### 3.1.2. Atomic Force Microscopy Analysis

Atomic force microscopy (AFM) was performed for analyzing the surface morphology of the scaffolds.

**Figure 4:**
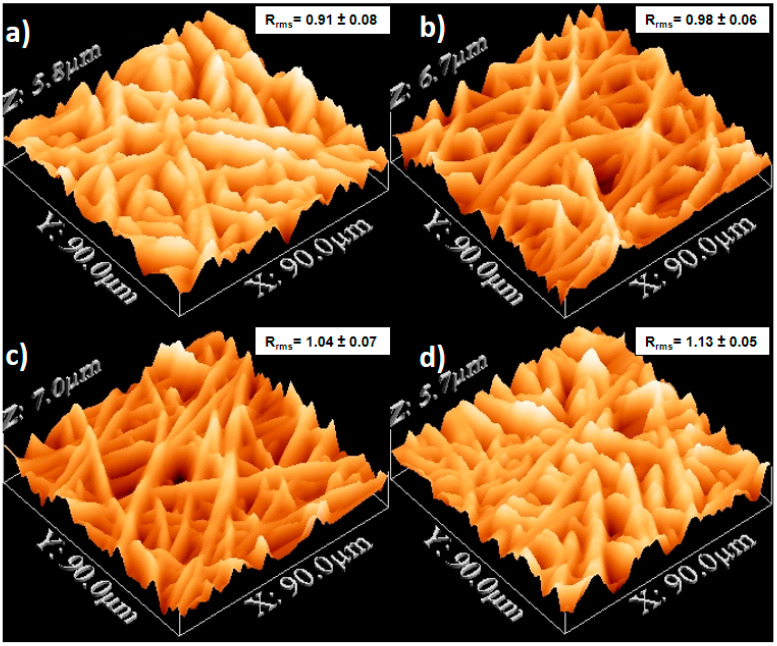
Atomic force microscopy (AFM) images of the electrospun PCL scaffolds prepared with voltages of: (**a**) 10 kV, (**b**) 15 kV, (**c**) 20 kV, and (**d**) 25 kV.

The surface roughness R_rms_ was obtained from samples at different voltages. The R_rms_ values of the electrospun PCL scaffolds were estimated from the AFM images (see Figure 4). For that, four regions of each sample were imaged. An increase in the R_rms_ value was obtained as voltage increases. Reported values of the R_rms_ were of 0.91 ± 0.08 µm and 1.13 ± 0.05 µm for the PCL scaffolds prepared at 10 kV and 25 kV, respectively.

This increment in the R_rms_ can be explained during the electrospinning process given that a turbulent flow is produced by increasing the voltage, which results in a rough surface of PCL scaffold. These morphologies generate different scaffolds architectures, which produces an increase in the R_rms_ of the nanofibers, Figure 2d.

Hassan et al. [9] found that the nanofibers’ diameter also affects the surface roughness, where a high roughness value was reported for larger fiber diameter of PCL nanofibers, which is in concordance with our work.

The roughness of the fiber scaffold plays an important role in the cellular response. A rough surface can promote better cells anchoring to the substrate and eventually improves the adsorption of proteins.

Zamani et al. [35] obtained higher cell adhesion on rough surfaces when they seeded cells from a nerve tissue cell line. They affirm that an increase in roughness promotes better adhesion of the nerve cells [35].

Some reports describe a greater proliferation of osteoblastic cells in rough surfaces, but it also reported that cell adhesion is dependent on the substrate characteristics and the type of cells to be evaluated [36].

#### 3.1.3. Analysis of the Surface Area and Pore Volume by BET Method

Studies of adsorption/desorption isotherms were carried out by means of the BET technique. Results of the surface area and total pore volume (empty spaces between nanofibers in 3D) of the scaffolds were obtained. Results can help to estimate the effect of the applied voltage on the morphology of the fibers.

**Figure 5:**
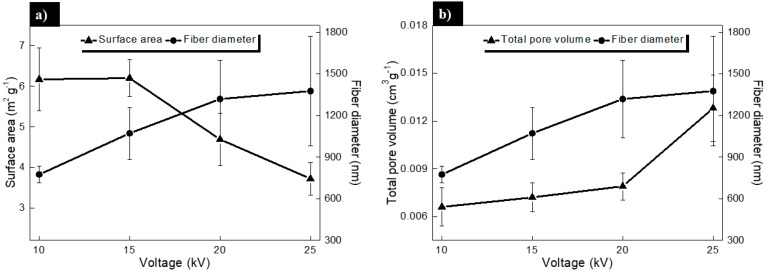
(**a**) Surface area and fiber diameter, and (**b**) total pore volume and fiber diameter of electrospun PCL nanofibers obtained at different voltage. In the Figure 5a (right side), total pore volume of the electrospun scaffolds at different voltages are presented. As the voltage increases, the total pore volume and the fiber diameter increases. In addition, the total pore volume increases by increasing the applied voltage, due to an increase in the fiber diameter. An increase in the pore volume indicates that the volume of air captured by the 3-D electrospun scaffolds is usually greater for fibers with large diameters [39].

It can be seen that surface area increases with increasing voltage, mainly due to the decreased fiber diameter (see Figure 5, left side). In this work, we observe that larger fiber diameters possess reduced surface area, which is consistent with the results reported by Pham et al. [34] and Deitzel et al. [37] related the surface area to volume ratio (specific surface area) with the radius of the fiber, obtaining a similar tendency reported by other authors [38].

By using mathematical models and experimental results, researchers have described relationships between the fiber diameter and the pore volume in 3-D electrospun scaffolds.

For example, Eichhorn et al. [33] used two variables: the fraction of fiber available for cell adhesion and the pore size and its distribution for growing cells. Their models predict that larger fiber diameter correlates with an increase in the pore size [33], in accordance with experimental data obtained for various polymers including PCL. Pham et al. [34] reported that a 2.5-fold increase in the diameter of electrospun PCL fibers results in a 2.25-fold increase in the average pore size.

The parameters exhibited in Figure 5 are of great relevance in tissue engineering, due to the controlled variables that could add functionality to the PCL scaffolds. Various reports [40,41] demonstrated that the design of the scaffold characteristics (fiber diameter, surface area, and pore volume) have a clear influence on cellular growth; therefore, an adequate conjugation of these variables could establish the biological regulation of the cells.

In this work, an interesting correlation of these characteristics was found between the fiber diameter and the total pore volume vs. applied voltage (see Figure 5b). It is known that fiber diameter dictates some surface properties such as the increment of pore size [42] and surface roughness [43] with increased fiber diameter in scaffold [44]. In this sense, Milleret et al. [45] reported that the scaffolds with smaller fibers induced smaller coagulation activation and less platelet adhesion when compared to scaffolds composed of larger fibers.

Authors mention that electrospinning parameters greatly affect fiber diameter together with the pore size and the overall scaffold roughness, but they can be adjusted for tissue engineering applications.

### 3.2. Physicochemical Characterization of the Electrospun PCL Scaffolds

#### 3.2.1. Fourier-Transform Infrared Spectroscopy in Attenuated Total Reflectance Mode

FTIR analysis was used to detect possible changes in the chemical structure of the PCL scaffolds during the electrospinning process. No significant changes were observed during the raw material (pellet) evaluation, or when the voltage was varied. Similar results were obtained by Kim et al. [46] for pure PCL (pellet).

**Figure 6:**
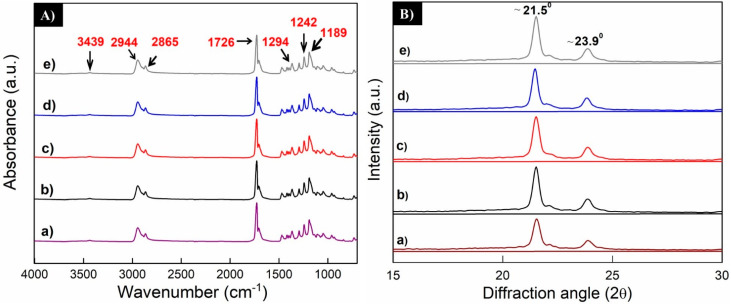
(**A**) FTIR-ATR spectra (left-side) and (**B**) diffractrograms (right-side) of electrospun PCL nanofibers obtained at different voltages: (**a**) pellet (raw material), (**b**) 10 kV, (**c**) 15 kV, (**d**) 20 kV, and (**e**) 25 kV.

As noted in Figure 6A, spectra exhibit the main absorption bands of the PCL. The asymmetric and symmetric stretching vibrations of the methylene groups (2944 cm^−1^ and 2865 cm^−1^, respectively); as well as the carbonyl group of esters (O-C=O) at 1726 cm^−1^. Additionally, a band at 1294 cm^−1^ corresponding to the C-O groups of the PCL crystalline phase was observed. Bands at 1418 cm^−1^, due to the bending of O-H linkage, 1242 cm^−1^, related to the asymmetric stretching of the C-O-C bonds and, 1189 cm^−1^, corresponding to the symmetric stretching of the OC-O bonds were detected [47].

Finally, it is possible to observe low intensity peaks at 984 cm^−1^, 961 cm^−1^, and 840 cm^−1^, which correspond to the rocking of the methylene groups [48]. In addition, the presence of a small band at 3439 cm^−1^ can be observed which is related to the O-H bond of the PCL end-groups.

#### 3.2.2. X-ray Diffraction

The X-ray diffraction patterns of the PCL scaffolds obtained at different voltage values are shown in Figure 6B. The presence of two main peaks around 21.5° and 23.9°, corresponding to (110) and (200) orientations of the orthorhombic crystalline structure are clearly distinguished [49,50]. Sharpening peaks indicate that samples belong to materials with considerable crystallinity index. No differences in the diffraction patterns were observed for prepared samples with different applied voltage, as well as for the PCL pellets. Crystallinity index values were similar for all the voltages (see Table 1). Thomas et al. [51] found that during the electrospinning process, as applied voltage (and the flow rate too) fiber formation, a stretching of fibers occurs so rapidly that the molecular chains of the PCL do not get enough time for complete crystallization.

#### 3.2.3. Contact Angle Measurements

Different parameters can influence the hydrophilic/hydrophobic character of the fibers such as the functional groups on the surface, the roughness, the polarity of the material, the orientation of the fibers, and wettability, which allow determining the degree of affinity of materials for polar or no-polar species. In this way, the contact angle analysis is a sensitive technique for determining the surface wettability of the materials [35]. Table 1 shows the results of the water contact angle measurements on the scaffolds obtained at different voltage values. PCL is known to possess highly hydrophobic characteristics.

Analysis of data shows that wetting of samples decreases with the applied voltage. It has been reported that values of contact angle in hydrophobic surfaces such as PCL, increases with surface roughness [52]. This report is similar to the results obtained in this work given that the R_rms_ value increases from 0.91 ± 0.08 µm to 1.13 ± 0.05 µm (see Figure 4), and the water contact angle increased from 125 ± 1° to 131 ± 1° (see Table 1), comparing the minor vs. the higher voltage. In tissue engineering, moderate hydrophilicity is preferred for cell adhesion to occur.

Therefore, this drawback could be overcome by incorporating polar functional groups on material surface, for example, by means of plasma treatments [39].

In addition, it is reported that a hydrophobic surface can be related with the presence of trapped air between the 3-D electrospun fibers. Hence, larger fiber diameters present more trapped air and therefore, a highly hydrophobic character [33,34,39].

### 3.3. Mechanical Characterization of PCL Electrospun Scaffolds

One of the most important aspects to be considered in scaffolds for tissue engineering are the mechanical properties. They are required to resist the in vivo applied physiological loads [44]. For skin regeneration, flexible materials are required, because they will be subjected to deformations when external forces are applied. Tensile tests are performed for soft tissues such as, ligaments, skin, cartilage, blood vessels, etc., whereas for hard tissues such as bone, the elastic modulus is the parameter employed [53]. Thus, the mechanical properties of the different scaffolds are normally obtained under tensile tests.

**Figure 7:**
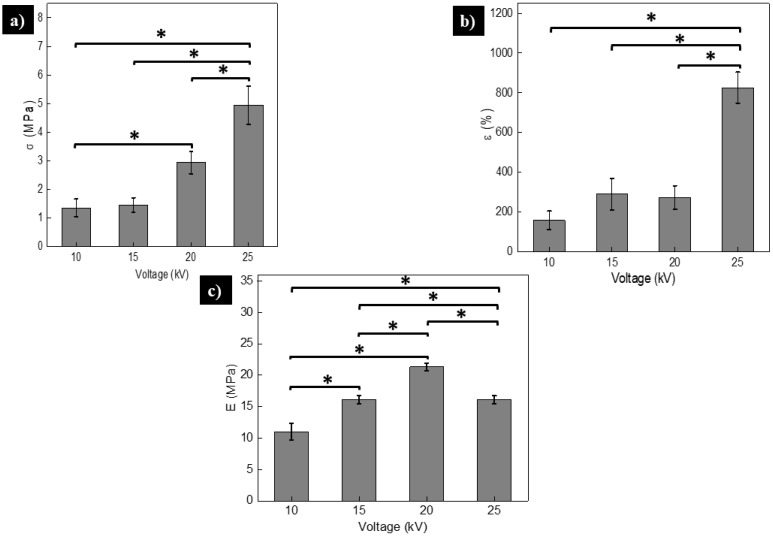
Mechanical properties of PCL scaffolds obtained at different voltages. (**a**) tensile strength (σ); (**b**) elongation (ε); and (**c**) elastic modulus (E). * Statistically significant changes were found in some cases. A value of *p* ≤ 0.05 was considered significant.

In Figure 7a, it can be observed that increasing the voltage increases the ultimate tensile strength (σ) of the scaffolds, but significant changes are found only in some cases: when the sample with highest voltage (25 kV) is compared with the others (10, 15 and/or 20 kV) and when the tensile strength values are compared with the scaffolds elaborated at 10 kV and 20 kV. This last one could contribute to an increase in tensile strength.

Because the expelled jet of polymer is randomly welded together, reaching the collector and causing the union of fibers to form stronger and more tensile strength connections that affect the morphology and the mechanical properties [54,55,56].

Nguyen et al. [57] reported that conglutination is common when high voltages are used in the electrospinning of scaffolds and allows increasing the tensile strength of the material due to the formation of a network of interlaced fibers. Additionally, Wei et al. [58] modeled the mechanical properties of an electrospun nanofibers network and they observed that conglutination of fibers leads to a significant increase in the tensile strength. These results are also consistent with the work of You et al. [59] who state that fiber fusion increases the tensile properties. These authors postulated that the increased tensile strength is attributed to the enhanced load transfer between fibers due to their fusion. The fiber-fiber load transfer occurs through the van der Waals interactions, as well as mechanical inter-locking, where introducing fusion points (by increasing the voltage) enhances the load transfer between the fibers and the tensile strength [59].

Figure 7b shows that elongation (ε) of the scaffolds increases with the applied voltage. In fact, significant changes were found when the ε values obtained at the highest voltage (25 kV) were compared with samples prepared with 10, 15 and/or 20 kV. It is important to mention that the ε values in the range of 200 to 800% are attractive in tissue engineering for soft tissues. The elongation values obtained in this work are similar to the values reported by Thomas et al. [51] for PCL scaffolds prepared by the electrospinning technique, and even higher than reported by other authors for electrospun PCL [10,60]. This can be interesting for applications requiring scaffolds with high deformation.

High values of the elastic modulus (E) are observed when samples are prepared with high voltage, except for 25 kV where the E value decreases (see Figure 7c). This can be due to the abrupt change in ε at this voltage (see Figure 7b). Substantial changes are observed for different cases (see Figure 7c).

Stylianopoulos et al. [61] demonstrated through computational calculations that the E values of electrospun scaffolds depend on the fiber diameter and their alignments. By using analytical models, the increment of the E value with the increased fiber diameter was predicted [62,63], although other authors affirm the contrary [64]. Berhan and Sastry [65] attributed the increment of the E value to fiber conglutination, rather than diameter. They demonstrated that the geometry of a welded fiber and the angle between two conglutinated fibers affects the forces of the network under applied load, in consistence with the Reneken et al. report [54]. In our work, this welded scaffold’s tendency was partially observed (Figure 2c,d).

**Figure 8:**
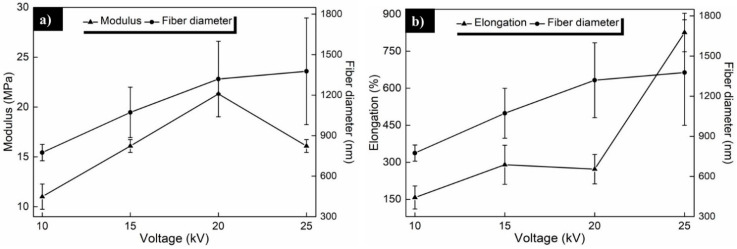
(**a**) Modulus and fiber diameter, and (**b**) elongation and fiber diameter vs. applied voltage of the obtained electrospun PCL fibers.

Figure 8a shows a modulus (E) and fiber diameter vs. applied voltage plot, where increased E and fiber diameter were observed with the applied voltage. In Figure 8b can be seen the elongation (ε) and fiber diameter vs. voltage plot, where ε and fiber diameter seem to increase with the applied voltage. In this last plot, the diameter and mechanical properties show a clear correlation with the voltage. From our results, we affirm that prepared PCL scaffolds with larger fiber diameters possess attractive mechanical properties that could be used for tissue engineering.

## 4. Conclusions

The effect of the applied voltage on the morphology and the mechanical properties of electrospun PCL scaffolds in the electrospinning process were studied. By increasing the applied voltage, larger fiber diameters can be produced. The morphology and the mechanical properties of electrospun PCL scaffolds were modified without affectation of their physicochemical properties. The crystalline structure of the prepared PCL fibers does not show modifications by changing the applied voltage. The fiber diameter, surface roughness, and total pores volume increase with the increased voltage. The tensile strength, elongation, and elastic modulus of the electrospun PCL were analyzed resulting in flexible and stronger fibers which are attractive for tissue engineering applications. Our results could be useful for further studies in which relationships between voltage and biological response of these PCL scaffolds are required to be used.

## Figures and Tables

**Table 1:** Crystallinity index, water contact angle and glass transition temperature (see Supplementary Materials) of electrospun nanofibers prepared at different voltages.

Parameters	Voltage (kV)			
	10	15	20	25
**Crystallinity Index (%)**	47	47	48	47
**Contact angle (°)**	125 ± 1 *	127 ± 2	130 ± 2	131 ± 1 *
**Glass Transition Temp (°C)**	−62	−61	−60	−60

* Statistical changes of the PCL scaffolds, by considering the low and high values. A value of *p* ≤ 0.05 was considered significant.

## Data Availability

Please Exclude.

## Supplementary Materials

The following are available online at https://www.mdpi.com/2073-4360/13/4/662/s1, Figure S1: Thermograms of PCL scaffolds obtained at different voltages. The glass transition temperature in all cases was around −61 ± 1 °C.
