# Supplementary material for: Morphological and Mechanical Properties of Electrospun Polycaprolactone Scaffolds: Effect of Applied Voltage
Source: Polymers (Basel). 2021 Feb 23;13(4):662. doi: 10.3390/polym13040662 (PMC7926916; doi:10.3390/polym13040662)
Supplement: Supplementary file 1 [file polymers-13-00662-s001.pdf]

polymers

Supplementary Materials

Morphological and Mechanical Properties of Electrospun Polycaprolactone Scaffolds: Effect of Applied Voltage

L.A. Can-Herrera [1,*], A.I. Oliva [1], M.A.A. Dzul-Cervantes [2], O.F. Pacheco-Salazar [2] and J.M. Cervantes-Uc [3]

[1] Departamento de Física Aplicada. CINVESTAV-IPN, Unidad Mérida, Carretera Antigua a Progreso Km. 6, Cordemex, C.P. 97310, Mérida, Yucatán, México; oliva@cinvestav.mx
[2] Instituto Tecnológico Superior de Calkiní en el Estado de Campeche. Av. Ah Canul S/N por Carretera Federal, C.P. 24900. Calkiní, Campeche, México; maadzul@itescam.edu.mx (M.A.A.D.-C.); ospacheco@itescam.edu.mx (O.F.P.-S.)
[3] Unidad de Materiales. Centro de Investigación Científica de Yucatán, A.C. Calle 43 No. 130 × 32 y 34, Chuburná de Hidalgo, C.P. 97205, Mérida, Yucatán, México; manceruc@cicy.mx
* Correspondence: luis.can.herrera.7@gmail.com; Tel.: +52-999-2430269

Citation: Can-Herrera, L.A.; Oliva, A.I.; Dzul-Cervantes, M.A.A.; Pacheco-Salazar, O.F.; Cervantes-Uc, J.M. Morphological and Mechanical Properties of Electrospun Polycaprolactone Scaffolds: Effect of Applied Voltage. *Polymers* **2021**, *13*, 662. https://doi.org/10.3390/polym13040662

Academic Editor: Sandrine Gerber-Lemaire

Received: 8 February 2021
Accepted: 20 February 2021
Published: 23 February 2021

Publisher's Note: MDPI stays neutral with regard to jurisdictional claims in published maps and institutional affiliations.

Copyright: © 2021 by the authors. Licensee MDPI, Basel, Switzerland. This article is an open access article distributed under the terms and conditions of the Creative Commons Attribution (CC BY) license (http://creativecommons.org/licenses/by/4.0/).

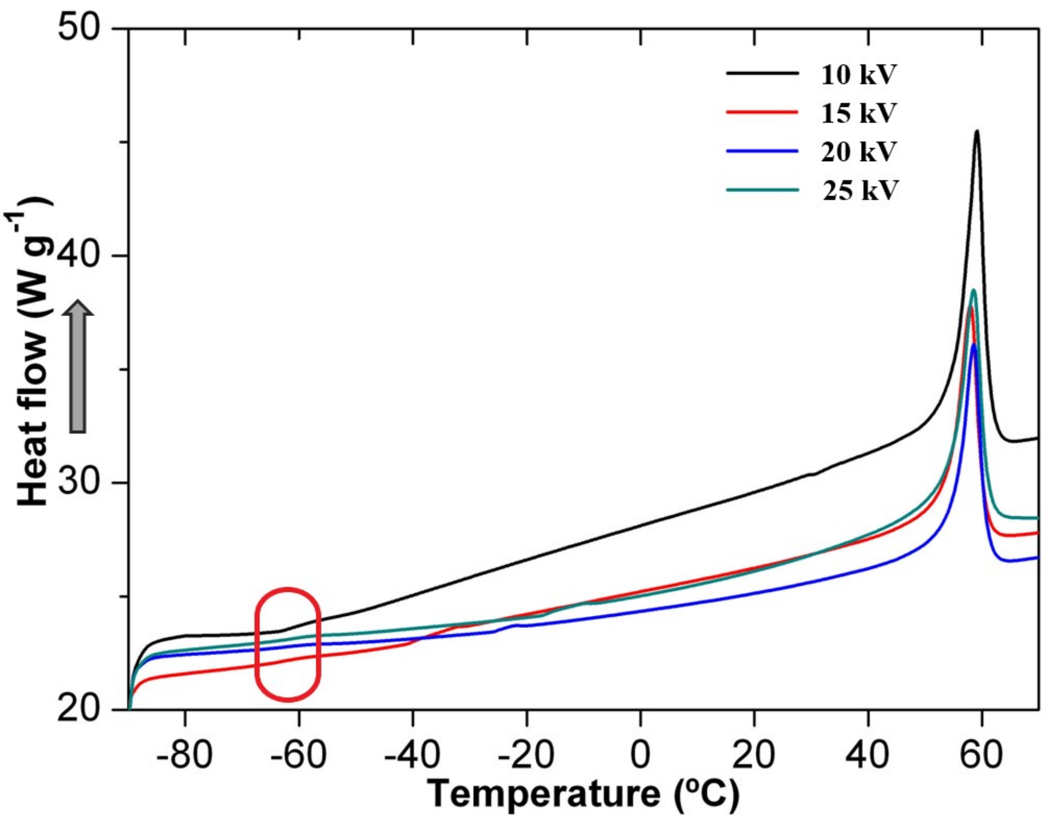

Figure 1. Thermograms of PCL scaffolds obtained at different voltages. The glass transition temperature in all cases was around −61 ± 1°C.
